# Development of Isocratic RP-HPLC Method for Separation and Quantification of L-Citrulline and L-Arginine in Watermelons

**DOI:** 10.1155/2018/4798530

**Published:** 2018-05-02

**Authors:** Rasdin Ridwan, Hairil Rashmizal Abdul Razak, Mohd Ilham Adenan, Wan Mazlina Md Saad

**Affiliations:** ^1^Centre of Medical Laboratory Technology, Faculty of Health Sciences, Universiti Teknologi MARA, Puncak Alam Campus, 42300 Bandar Puncak Alam, Selangor, Malaysia; ^2^Centre of Medical Imaging, Faculty of Health Sciences, Universiti Teknologi MARA, Puncak Alam Campus, 42300 Bandar Puncak Alam, Selangor, Malaysia; ^3^Faculty of Applied Sciences, Universiti Teknologi MARA, 40450 Shah Alam, Selangor, Malaysia; ^4^Atta-ur-Rahman Institute for Natural Product Discovery, Level 9, Bangunan FF3, Universiti Teknologi MARA, Puncak Alam Campus, 42300 Bandar Puncak Alam, Selangor, Malaysia

## Abstract

Watermelons* (Citrullus lanatus)* are known to have sufficient amino acid content. In this study, watermelons grown and consumed in Malaysia were investigated for their amino acid content, L-citrulline and L-arginine, by the isocratic RP-HPLC method. Flesh and rind watermelons were juiced, and freeze-dried samples were used for separation and quantification of L-citrulline and L-arginine. Three different mobile phases, 0.7% H_3_P0_4_, 0.1% H_3_P0_4_, and 0.7% H_3_P0_4_ : ACN (90 : 10), were tested on two different columns using Zorbax Eclipse XDB-C_18_ and Gemini C_18_ with a flow rate of 0.5 mL/min and a detection wavelength at 195 nm. Efficient separation with reproducible resolution of L-citrulline and L-arginine was achieved using 0.1% H_3_P0_4_ on the Gemini C_18_ column. The method was validated and good linearity of L-citrulline and L-arginine was obtained with *R*^2^ = 0.9956, *y* = 0.1664*x* + 2.4142 and *R*^2^ = 0.9912, *y* = 0.4100*x* + 3.4850, respectively. L-citrulline content showed the highest concentration in red watermelon of flesh and rind juice extract (43.81 mg/g and 45.02 mg/g), whereas L-arginine concentration was lower than L-citrulline, ranging from 3.39 to 11.14 mg/g. The isocratic RP-HPLC method with 0.1% H_3_P0_4_ on the Gemini C_18_ column proved to be efficient for separation and quantification of L-citrulline and L-arginine in watermelons.

## 1. Introduction

*Citrullus lanatus* (Thunb.) Matsum. and Nakai, commonly known as watermelon, is a nonseasonal fruit which is cultivated abundantly in Malaysia and other tropical regions [1]. It belongs to the Cucurbitaceae plant family, which originated from the African Kalahari Desert [1]. Watermelons have high content of phytonutrients and are rich in dietary antioxidants such as carotenoids (lycopene and *β*-carotene), polyphenolics, ascorbic acid, and significant amino acids [2]. Watermelons are usually consumed by juicing the flesh, beneficial in the prevention and improvement of health problems, such as cardiovascular diseases, erectile dysfunction, hypertension, and cancers [3]. Figueroa et al. [4] demonstrated that watermelon juice supplementation improves aortic hemodynamics by reducing the reflected wave amplitude in prehypertensive individuals. A study by Poduri et al. [5] reported that watermelon attenuated hypercholesterolemia-induced atherosclerosis in mice. Commercial watermelon juices provide enormous marketing potential and nutritious drinks for individuals to maintain a healthy lifestyle.

**Figure 1:**
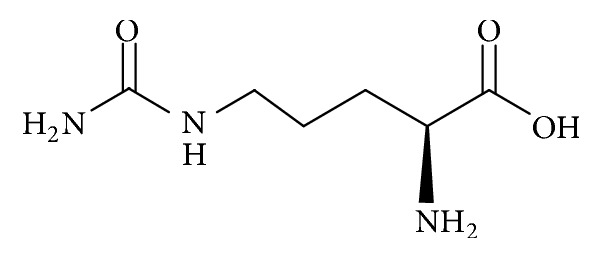
Molecular structure of L-citrulline (175.2 g/mol).

**Figure 2:**
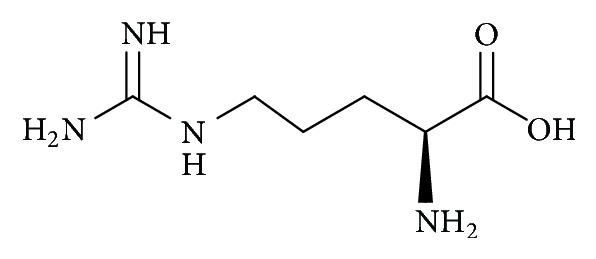
Molecular structure of L-arginine (174.2 g/mol).

Amino acids, particularly L-citrulline and L-arginine, are regarded as major types of phytonutrients present in watermelons which may contribute to their reputed and diversified health benefits [6]. L-citrulline, C_6_H_13_N_3_O_3_ (IUPAC name: 2-amino-5-(carbamoylamino)pentanoic acid) (Figure 1), is a nonessential amino acid firstly identified from watermelon,* Citrullus vulgaris* Schrad. [7, 8]. L-citrulline is a physiological endogenous amino acid to most living systems involved in protein metabolism and removal of excess metabolic ammonia [9]. It serves as a precursor for L-arginine and product of nitric oxide (NO) cycle [10]. L-arginine, C_6_H_14_N_4_O_2_ (IUPAC name: (*S*)-2-amino-5-guanidinopentanoic acid) (Figure 2), is a semiessential and free form physiological amino acid that functions as one of 20 building block proteins for biological processes such as cell division, ammonia removal, wound healing, and hormone release [7, 8]. Wu et al. [11] demonstrated that supplementation of L-citrulline and L-arginine from watermelon juice improved serum levels of NO metabolites and aortic endothelial-mediated vasodilation in diabetic rats.

L-citrulline and L-arginine are present in all parts of watermelon fruits including flesh, rind, and seed [7]. A study done by Rimando and Perkins-Veazie [12] reported that the rind of red watermelon and yellow watermelon contains more L-citrulline at a concentration ranging from 15.6 to 29.4 mg/g than flesh, 7.9–28.5 mg/g. Similar to the above finding, Jayaprakasha et al. [13] reported that rinds of* Citrullus vulgaris* varieties such as petite treat and jamboree watermelon and also yellow crimson watermelon contained slightly higher L-citrulline ranging from 13.95 to 28.46 mg/g than flesh, 11.25–16.73 mg/g. These findings suggested that watermelon rind has an abundance of L-citrulline content in comparison to its content in flesh.

Analyses of L-citrulline and L-arginine were routinely conducted using capillary electrophoresis and quantification by a spectrophotometric method; however, the method is less sensitive, leading to discrepancies in the outcomes [6]. L-citrulline and L-arginine are polar, nonvolatile, and devoid of chromophores; thus analysis by reverse-phase high performance liquid chromatography (RP-HPLC) commonly employed a derivatization method using pre- or postcolumn derivatization [14–17]. Jayaprakasha et al. [13] stated that precolumn derivatization such as orthophthalaldehyde (OPA), naphthalene-2,3-dicarboxaldehyde, or 4-dimethylaminoazobenzene-4′-sulfonyl chloride (dabsyl chloride) was able to provide accurate and stable chromatography baseline, but the reactions were unstable and affected by the sample matrix [18]. Postcolumn derivatization by ninhydrin is tedious due to long analysis time up to 72 hours and instability of derivatization reagents that may cause poor compound recovery [19]. Analysis of underivatized L-citrulline and L-arginine is warranted for rapid and effective quantification of these compounds. Given that no amino acids content of L-citrulline and L-arginine in Malaysia watermelons has been reported so far, we have developed an isocratic RP-HPLC method for separation and quantification of L-citrulline and L-arginine in watermelons.

## 2. Materials and Methods

### 2.1. Chemicals and Reagents

L-citrulline (purity ≥ 99%) and L-arginine (purity ≥ 98%) standard were purchased from Sigma-Aldrich (St. Louis, MO, USA). Methanol and acetonitrile of HPLC grade were purchased from Merck (Germany). Phosphoric acid (purity ≥ 85%) was purchased from Sigma-Aldrich (St. Louis, MO, USA). Deionized water was prepared using ultrapure water purifier system (Elgastat, Bucks, UK).

### 2.2. Instrumentation

The isocratic RP-HPLC method was carried out using Thermo Scientific™ Dionex-UltiMate™ 3000 HPLC system equipped with solvent reservoirs, LPG-3400SD pump, WPS-3000 autosampler injector, TCC-3000 column oven, and DAD-3000 ultraviolet-visible (UV-Vis) diode array detector module operated at four wavelengths per analysis. Chromeleon data software (Version 7) was used for data analysis.

### 2.3. Sample Preparation

*Citrullus lanatus* (Thunb.) Matsum. & Nakai of red watermelon and yellow crimson watermelon was obtained from Selangor Fruit Valley, Selangor. Seeds were removed manually and the edible part was cut into cubes. Watermelon flesh and rind were juiced and frozen at −80°C for at least 2 days. The frozen juices were put in a freeze-drier (Labconco, USA) for 4 days until completely dried. The dried juice powders were kept at −20°C. For the analysis, samples were prepared in the form of juice extract and methanol extract. Juice extract was prepared directly by dissolving the dried juice powder in dH_2_O. For methanol extract, a known quantity of dried juice powders was extracted with 30 mL of MeOH and 1 mL of 1 N HCl, vortexed, and sonicated for 30 minutes. The samples were macerated by cold maceration for a period of 72 hours in an orbital shaker. Methanol extracts were then filtered using Whatman filter paper. The residues were reextracted twice using fresh solvent and the three methanol extracts were pooled. The obtained methanol extracts were evaporated to dryness using a rotary vacuum evaporator at 60°C and stored at 4°C until analysis.

### 2.4. Isocratic RP-HPLC Analysis

#### 2.4.1. Standard Preparation Procedure

A stock solution of L-citrulline and L-arginine was prepared individually in dH_2_O at 1 mg/mL and filtered through a 0.45 *μ*m syringe filter (Bioflow). A mixed standard solution was prepared by mixing an equal volume of each standard stock solution. A series of working standard solutions was prepared by diluting the stock solution with dH_2_O in the range of 0.1–1000 *μ*g/mL.

#### 2.4.2. Sample Preparation Procedure

Juice extracts were prepared directly by dissolving the dried juices powder in dH_2_O at 5 mg/mL. Crude methanol extracts were also dissolved in dH_2_O at 5 mg/mL and vortexed for 15 minutes. All extracts were filtered through 0.45 *μ*m filters and injected to isocratic RP-HPLC.

#### 2.4.3. Chromatographic Analysis

Preliminarily, three different concentrations of ion-pair reagents, phosphoric acid (H_3_P0_4_), or addition of acetonitrile (ACN) as mobile phases, 0.7% H_3_P0_4_, 0.1% H_3_P0_4_, and 0.7% H_3_P0_4_ : ACN (90 : 10) (Table 1), were tested for separation and determination of L-citrulline and L-arginine standard. The column temperature was fixed at room temperature and UV-Vis detection was performed at 195 nm. The RP-HPLC columns [i.e., Zorbax Eclipse XDB-C_18_, 250 mm × 4.6 mm, 80 Å, 5 *μ*m (Phenomenex, Torrance, CA), and Gemini C_18_, 250 × 4.6 mm, 110 Å, 3 *μ*m (Phenomenex, Torrance, CA)] were used. The analysis proceeded for quantification of both compounds, L-citrulline and L-arginine in watermelons juice extracts and methanol extracts using the chosen column and mobile phase: Gemini C_18_ eluted by 0.1% H_3_P0_4_ with a flow rate of 0.5 mL/min at 195 nm. Chromeleon software was used for quantification of L-citrulline and L-arginine. The concentration of L-citrulline and L-arginine content was quantified based on the linear curve of standards. The content of compounds was expressed as milligrams per gram (mg/g) of sample extracts.

#### 2.4.4. Method Validation

The validation of the isocratic RP-HPLC method was performed for linearity of calibration curve, limit of detection (LOD), limit of quantification (LOQ), accuracy, and precision. The linearity of the isocratic RP-HPLC method for quantification of compounds was constructed using the concentration range of 0.1–1000 *μ*g/mL for L-citrulline and 0.1–500 *μ*g/mL for L-arginine. The regression equation was calculated in the form of *y* = *ax* + *b*, where *x* is the concentration and *y* is the peak area of compounds. Linearity was established by the coefficient of determination (*R*^2^). LOD and LOQ were measured based on signal-to-noise ratio (S/N) method. LOD is the lowest concentration of analyte that can be detected with signal-to-noise ratio of 3 : 1 and LOQ is the lowest concentration that can be quantified with acceptable precision and accuracy with signal-to-noise ratio of 10 : 1. S/N of 3 is considered acceptable for LOD, while LOQ is established at S/N of 10. Precision of the method was determined as percentage relative standard deviation (%RSD) of peak area of intraday and interday analysis data. Intraday (three times in a day operation under the same conditions) and interday (three different days) studies were performed at three different concentrations (Level 1: 20 *μ*g/mL; Level 2: 60 *μ*g/mL; Level 3: 150 *μ*g/mL). The resulting peak area was used to calculate SD and the relative standard deviation (%RSD). Accuracy of the method by recovery study was done by adding a known amount of reference standard solution (three concentrations) to test samples. The spiked extract solutions were injected three times, and the recovery was calculated with the value of detected versus added amounts.

## 3. Results and Discussion

### 3.1. Separation of L-Citrulline and L-Arginine by Isocratic RP-HPLC Method

The initial isocratic RP-HPLC method for separation of mixed standard, L-citrulline, and L-arginine was performed using selected mobile phases according to previous literatures with slight modifications [8, 13, 20]. Interaction between mobile phase and stationary phases in isocratic RP-HPLC is important for the determination of solutes' retention time [21].

**Figure 3:**
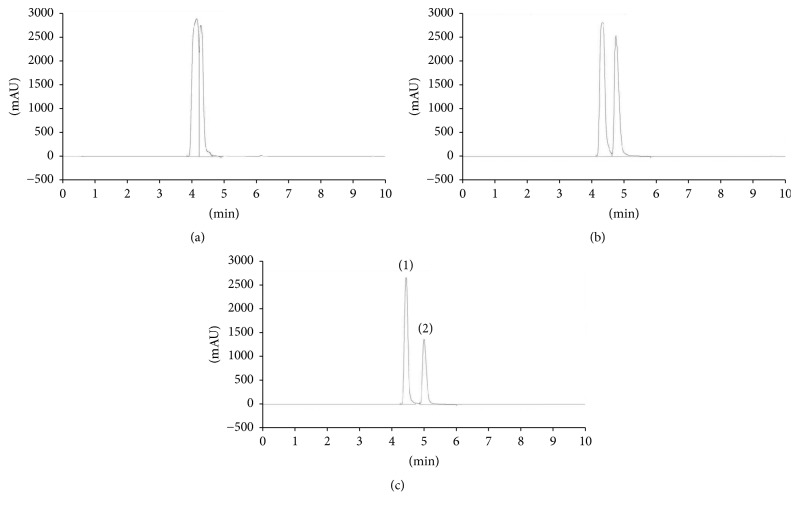
Comparative chromatograms showing isocratic RP-HPLC separation of mixed standard, L-citrulline, and L-arginine in different mobile phases: (a) 0.7% H_3_P0_4_ : ACN (90 : 10); L-citrulline and L-arginine were unretained and coeluted at *k* value close to zero; (b) 0.7% H_3_P0_4_; L-citrulline and L-arginine were slightly retained and partially separated; (c) 0.1% H_3_P0_4_; L-citrulline and L-arginine were efficiently separated with reproducible peaks. The peaks marked represent (1) L-arginine and (2) L-citrulline.

In this study, separation for determination of mixed standard, L-citrulline, and L-arginine was performed using a hydrophilic anionic ion-pairing reagent with different concentrations of phosphoric acid (H_3_P0_4_) or addition of acetonitrile (ACN) as mobile phases: 0.7% H_3_P0_4_, 0.1% H_3_P0_4_, and 0.7% H_3_P0_4_ : ACN (90 : 10). The mobile phase at the concentration of 0.7% H_3_P0_4_ : ACN (90 : 10) resulted in L-citrulline and L-arginine were unretained and coeluted (*k* value close to 0) as shown in Figure 3(a). The mixture of 0.7% H_3_P0_4_ : ACN (90 : 10) is highly hydrophilic, leading to rapid elution of L-citrulline and L-arginine with poor separation. Peaks of L-citrulline and L-arginine were slightly retained and partially separated using 0.7% H_3_P0_4_ (Figure 3(b)). However, optimum resolution was not achieved by 0.7% H_3_P0_4_ as *k* value between L-citrulline and L-arginine is close to 1. The mobile phase of 0.1% H_3_P0_4_ resulted in efficient separation with reproducible peaks of L-citrulline and L-arginine although all chromatograms showed stable baseline (Figure 3(c)). This finding is in agreement with Fekete et al. [22] who noted that 0.1% H_3_P0_4_ acts as a good separation agent by increasing the polarity and improving the retention time of zwitterionic molecules including amino acids. Dolan [23] supported the notion that 0.1% H_3_P0_4_ adequately provides reasonable buffering for amino acids separation by RP-HPLC. This showed that a concentration less than 1.0% H_3_P0_4_ as mobile phase provides efficient separation of amino acids, peptides, or proteins as demonstrated by Shibue et al. [24]. Thus, the mobile phase 0.1% H_3_P0_4_ is proven to provide efficient separation and the best resolution of mixed standard, L-citrulline, and L-arginine.

**Figure 4:**
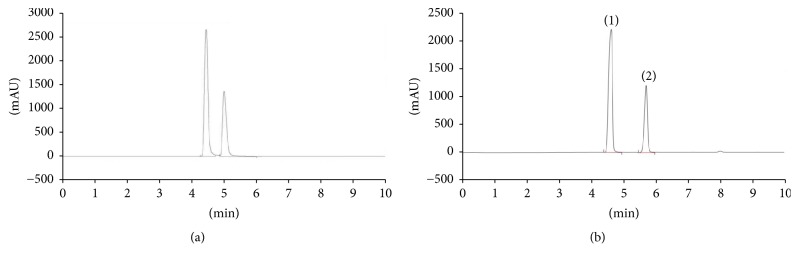
Comparative chromatograms showing isocratic RP-HPLC separation of mixed standard, L-citrulline, and L-arginine from 2 different columns: (a) Zorbax Eclipse XDB-C_18_, 5 *μ*m, and (b) Gemini C_18_, 3 *μ*m; efficient separation and the best resolution were achieved by the Gemini C_18_ column which showed that compounds are well separated. The peaks marked represent (1) L-arginine and (2) L-citrulline.

**Figure 5:**
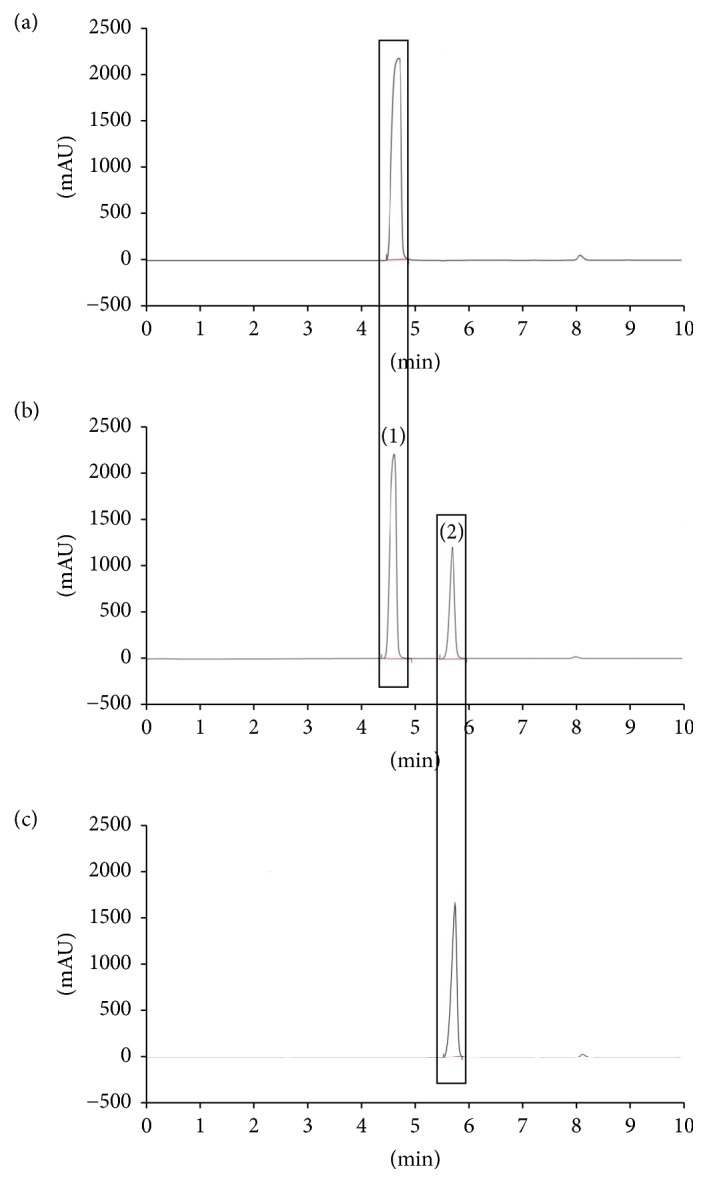
Comparative chromatograms showing isocratic RP-HPLC separation of individual and mixed standard, L-citrulline, and L-arginine using Gemini C_18_: (a) L-arginine, (b) mixed standard, and (c) L-citrulline. The peaks marked represent (1) L-arginine and (2) L-citrulline.

The study also evaluated separation of mixed standard in two different columns, Zorbax Eclipse XDB-C_18_ and Gemini C_18_ using 0.1% H_3_P0_4_. Zorbax Eclipse XDB-C_18_ did not provide good separation and resolution of L-citrulline and L-arginine as shown in Figure 4(a). A study by Barber and Joseph [25] showed that polar compounds were less separated and not well resolved using Zorbax Eclipse XDB-C_18_ with a longer analysis time of 54 minutes. Efficient separation and resolution of L-citrulline and L-arginine from mixed standard were achieved using Gemini C_18_ as shown in Figure 4(b). L-citrulline and L-arginine are eluted at a short retention time with L-arginine, 4.773 min, followed by L-citrulline at 5.787 min (Figure 5). Efficient separation of L-citrulline with a retention time of about 4 min was achieved on the Gemini C_18_ column due to the high degree similarity of column with polar compounds [13]. Gemini C_18_ is a new generation hybrid column end-capped with porous silica as base core and polymer media coated on top of the silica core which exhibit silica-like mechanical properties of base material while similarly decreasing the number of residual silanols [26]. This result demonstrated that Gemini C_18_ is the most suited column for efficient separation of mixed standard, L-citrulline and L-arginine.

The result from chromatography separation of L-citrulline and L-arginine shown in Figure 5(b) demonstrated that the reverse-phase (RP) mode provided efficient separation and substantial retention achieved on both polar compounds without the need for derivatization. RP mode can efficiently be applied in this study although Brown et al. [27] proposed the use of hydrophilic interaction liquid chromatography (HILIC) mode after cyano- and pentafluorophenyl-propyl stationary phases failed to retain target compounds including L-citrulline and L-arginine. HILIC mode is primarily used when separation of very polar compounds is needed or for incomplete chromatographic separation in RP mode [28]. However, HILIC mode required an expensive and robust system equipped with tandem mass spectrometric (MS) detection for L-citrulline and L-arginine separation in high biological matrix samples such as serum and plasma [16, 27]. Complete chromatographic separation of L-citrulline and L-arginine by RP mode in this study eliminates the use of HILIC mode.

### 3.2. Validation of Isocratic RP-HPLC Method

Method validation of isocratic RP-HPLC method was performed by determination of linearity, LOD, LOQ, recovery, and intraday and interday analysis. The results of linearity, LOD, and LOQ were summarized in Table 2. Good linear regression equations of L-citrulline and L-arginine standard are displayed between corresponding peak areas versus concentrations of compounds based on the correlation coefficients (*R*^2^ = 0.9956, *y* = 0.1664*x* + 2.4142 and *R*^2^ = 0.9912, *y* = 0.4100*x* + 3.4850, resp.). The LOD and LOQ for L-citrulline were 0.42 *μ*g/mL and 1.28 *μ*g/mL while those for L-arginine were 0.88 *μ*g/mL and 2.66 *μ*g/mL, which demonstrated that isocratic RP-HPLC method was efficiently sensitive. The method had good accuracy that showed efficient recoveries for both compounds ranging from 98.88% to 103.41% (Table 3). The RSD (%) for intraday and interday precision ranged from 0.37% to 1.09% for L-citrulline and 0.33% to 2.05% for L-arginine, in which both RSD ≤ 2% (Table 4). These validation results confirmed that the isocratic RP-HPLC method is precise, accurate, and sensitive for simultaneous quantification of L-citrulline and L-arginine.

### 3.3. Quantification of L-Citrulline and L-Arginine Contents in Two Different Watermelon Extracts

**Figure 6:**
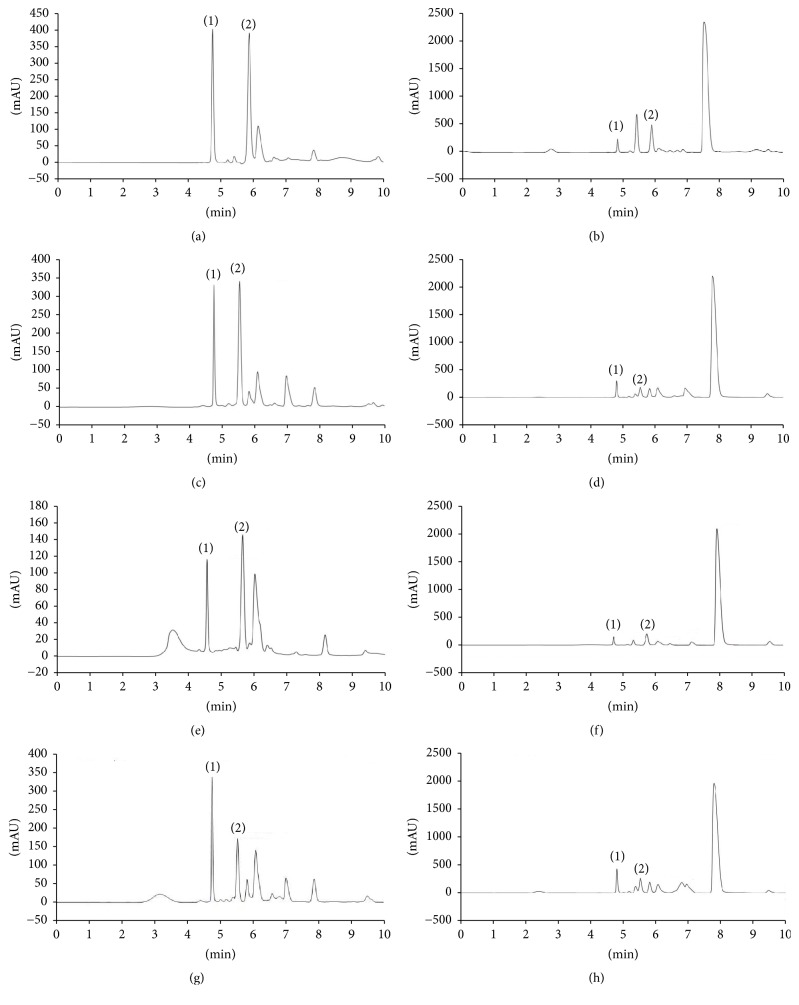
Comparative chromatographic profiles showing isocratic RP-HPLC separation of flesh and rind from juice extracts and methanol extracts of red watermelon and yellow crimson watermelon using Gemini C_18_. Red watermelon: (a) flesh juice extract, (b) rind juice extract, (c) crude flesh extract, and (d) crude rind extract. Yellow crimson watermelon: (e) flesh juice extract, (f) rind juice extract, (g) crude flesh extract, and (h) crude rind extract. The peaks marked represent (1) L-arginine and (2) L-citrulline.

Consumption of watermelon extracts rich in L-citrulline and L-arginine is proven to be beneficial for diseases prevention. Thus, a rapid, reliable, and efficient isocratic RP-HPLC method is essential for simultaneous quantification of these amino acids in juice extracts and methanol extracts. The chromatographic profiles of both extracts in red watermelon and yellow crimson watermelon are presented in Figure 6. Quantification of L-citrulline and L-arginine was performed using Chromeleon software. The content was calculated based on the calibration curve of L-citrulline and L-arginine standard achieved with good correlation coefficients and linear regression equations, *R*^2^ = 0.9956, *y* = 0.1664*x* + 2.4142 and *R*^2^ = 0.9912, *y* = 0.4100*x* + 3.4850, respectively. The results are tabulated in Table 5.

Red watermelon juice extract showed slightly high yield of L-citrulline in rind, 45.02 mg/g compared to flesh, 43.81 mg/g. Similar trends were shown in L-citrulline content in rind and flesh of yellow crimson juice extract, 16.61 mg/g and 15.77 mg/g, respectively. This finding is in accordance with the study by Jayaprakasha et al. [13] which found that rinds from* C. vulgaris* watermelon varieties of petite treat and jamboree watermelon and yellow crimson watermelon contained significantly high L-citrulline, 13.95 mg/g, 20.84 mg/g, and 28.46 mg/g, respectively, compared to flesh, 11.25 mg/g, 16.73 mg/g, and 14.74 mg/g, respectively, using RP-HPLC method. L-arginine content in red watermelon juice extract was higher in flesh, 11.10 mg/g, compared to rind, 3.39 mg/g. L-arginine content was approximately 3-fold lower than L-citrulline in red watermelon flesh juice extract. Consumption of watermelon flesh juice extract aided in efficient conversion of significantly high L-citrulline, a potent endogenous precursor to L-arginine in the kidney, which resulted in increased plasma L-arginine concentration. Findings by Collins et al. [29] proved that plasma L-arginine concentration increased by 95.2 ± 3.5 *μ*M and 108.0 ± 4.1 *μ*M compared to normal plasma baseline, 86.4 ± 3.5 *μ*M after 3 weeks of consumption of 780 mL (~1 g L-citrulline/day) to 1560 mL (~2 g L-citrulline/day) of watermelon juices. Recently, a study by Bailey et al. [30] supported the notion of the increased plasma L-arginine concentration by 116 ± 9 *μ*M compared to placebo, 67 ± 13 *μ*M after 2 weeks of 300 mL/day watermelon juice consumption, which contains ~3.4 g L-citrulline/day. L-citrulline content in crude flesh and rind extract of red watermelon and yellow crimson watermelon varied in the range of 13.91–24.99 mg/g, while L-arginine content was in the range of 4.08–8.41 mg/g. L-citrulline and L-arginine content is much lower in methanol extracts compared to juice extracts. Fish and Bruton [17] stated that methanol extracts may diminish the solubility of amino acids, thus marked reduction in amino acids yield. The quantitative results confirmed that watermelon juice extracts most effectively quantified higher yield of L-citrulline and L-arginine, and this study's outcomes may possibly suggest that juice extraction method is best in optimizing amino acids yield.

## 4. Conclusion

The isocratic RP-HPLC method has been successfully developed for separation and quantification of L-citrulline and L-arginine content in both watermelon extracts of flesh and rind using the selected mobile phase (0.1% H_3_P0_4_) in Gemini C_18_. The established isocratic RP-HPLC method provides evidence that L-citrulline and L-arginine are best retained using Gemini C_18_ column. The validated method is robust, sensitive, accurate, and precise with good linearity (*R*^2^ ≥ 0.99), low values of LOD and LOQ, recoveries within 98.88%–103.41%, and %RSD precision less than 2%. Juice extract effectively yielded higher L-citrulline and L-arginine content by juice extraction method; thus it is potentially used for quantitative amino acids analysis. The present study procedure may provide a basis for separation and quantification of L-citrulline and L-arginine in local watermelons. The high content of L-citrulline and L-arginine suggested watermelons as a good source of nutraceutical and health benefits ingredients. However, further researches are necessary to explore biological activities such as aphrodisiac properties of these active constituents in watermelons to support their potential in human diet and prevention of health related diseases.

## Figures and Tables

**Table 1:** Selection of mobile phases for separation of mixed standard, L-citrulline, and L-arginine by isocratic RP-HPLC.

Mobile phase	Ratio	Solution mixture (%)
0.7% H_3_P0_4_	100	0.7% H_3_P0_4_ + 99.3% dH_2_O
0.1% H_3_P0_4_	100	0.1% H_3_P0_4_ + 99.9% dH_2_O
0.7% H_3_P0_4_ : ACN	90 : 10	(0.7% H_3_P0_4_ + 99.3% dH_2_O) + 100% ACN

**Table 2:** Calibration data of L-citrulline and L-arginine standard reported from isocratic RP-HPLC method.

Standard	Concentration range (*µ*g/mL)	Regression equation	Correlation coefficient (*R*^2^)	Limit of detection (*µ*g/mL)	Limit of quantification (*µ*g/mL)
L-citrulline	0.01–1000	*y* = 0.1664*x* + 2.4142	0.9956	0.42	1.28
L-arginine	0.01–500	*y* = 0.4100*x* + 3.4850	0.9912	0.88	2.66

**Table 3:** Recovery of L-citrulline and L-arginine standard reported from isocratic RP-HPLC method.

Compounds	Added concentration (*µ*g/mL)	Measured concentration (*µ*g/mL)	Recovery (%)	RSD (%)
L-citrulline	100	101.94	101.94	1.70
	60	61.24	102.07	1.46
	30	31.02	103.38	1.00

L-arginine	100	99.87	99.87	1.96
	60	59.33	98.88	1.76
	30	31.02	103.41	1.17

**Table 4:** Intraday and interday analysis of L-citrulline and L-arginine standard reported from isocratic RP-HPLC method.

Compounds	Concentration (*µ*g/mL)	Intraday (*n* = 3) (%)		Interday (*n* = 3) (%)	
		Mean	RSD	Mean	RSD
L-citrulline	150	101.25	1.23	100.42	1.09
	60	102.07	1.46	103.44	1.01
	20	116.44	2.03	116.22	0.37

L-arginine	150	102.01	0.56	103.83	2.05
	60	96.26	1.04	97.36	1.13
	20	95.41	0.68	95.94	0.33

**Table 5:** L-citrulline and L-arginine contents (mg/g) in juice extract and methanol extract of watermelon flesh and rind juices.

Watermelon	Compounds	Juices	Juice extract (mg/g)	Methanol extract (mg/g)
Red	L-citrulline	Flesh	43.81	16.22
		Rind	45.02	24.99
	L-arginine	Flesh	11.10	6.42
		Rind	3.39	4.08

Yellow crimson	L-citrulline	Flesh	15.77	13.91
		Rind	16.61	16.03
	L-arginine	Flesh	8.23	6.68
		Rind	11.14	8.41
